# Effective Assessments of a Short-Duration Poor Posture on Upper Limb Muscle Fatigue Before Physical Exercise

**DOI:** 10.3389/fphys.2020.541974

**Published:** 2020-10-06

**Authors:** Lei Lu, Mark Robinson, Ying Tan, Kusal Goonewardena, Xinliang Guo, Iven Mareels, Denny Oetomo

**Affiliations:** ^1^Melbourne School of Engineering, The University of Melbourne, Parkville, VIC, Australia; ^2^School of Mechatronics Engineering, Harbin Institute of Technology, Harbin, China; ^3^Elite Akademy Sports Medicine, Parkville, VIC, Australia; ^4^IBM Research - Australia, Southbank, VIC, Australia

**Keywords:** poor posture, surface electromyography, muscle fatigue, frequency spectrum, physical activity

## Abstract

A forward head and rounded shoulder posture is a poor posture that is widely seen in everyday life. It is known that sitting in such a poor posture with long hours will bring health issues such as muscle pain. However, it is not known whether sitting in this poor posture for a short period of time will affect human activities. This paper investigates the effects of a short-duration poor posture before some typical physical activities such as push-ups. The experiments are set up as follows. Fourteen male subjects are asked to do push-ups until fatigue with two surface electromyography (sEMG) at the upper limb. Two days later, they are asked to sit in this poor posture for 15 min with eight sEMG sensors located at given back muscles. Then they do the push-ups after the short-duration poor posture. The observations from the median frequency of sEMG signals at the upper limb indicate that the short-duration poor posture does affect the fatigue procedure of push-ups. A significant decreasing trend of the performance of push-ups is obtained after sitting in this poor posture. Such effects indicate that some parts of the back muscles indeed get fatigued with only 15 min sitting in this poor posture. By further investigating the time-frequency components of sEMG of back muscles, it is observed that the low and middle frequencies of sEMG signals from the infraspinatus muscle of the dominant side are demonstrated to be more prone to fatigue with the poor posture. Although this study focuses only on push-ups, similar experiments can be arranged for other physical exercises as well. This study provides new insights into the effect of a short-duration poor posture before physical activities. These insights can be used to guide athletes to pay attention to postures before physical activities to improve performance and reduce the risk of injury.

## 1. Introduction

Poor posture is regarded as prolonged deviations from the neutral spine, and it usually can be characterized by the forward head posture, rounded shoulders, and increased thoracic kyphosis (Wong and Wong, 2008; Singla and Veqar, 2017). Due to bad habits, poor posture is commonly observed in various scenarios in daily life. It is reported that poor posture presents emerging health risks (Jia and Nussbaum, 2018), especially, the prolonged poor posture is usually associated with the use of smartphones and other portable devices, which are reported having increasing musculoskeletal problems (Jung et al., 2016).

It is reported that prolonged poor posture may result in body discomfort and myofascial pain syndrome by placing stress and excessive tension on the lumbar vertebrae (Swann, 2009). For example, the forward head posture affected neck extensor muscle thickness (Goodarzi et al., 2018), and the spinal misalignment had bad influence on back muscle strength and shoulder range of motion (Imagama et al., 2014). Meanwhile, some other studies showed that poor posture had negative impact on the musculo-skeletal system, caused localized muscle fatigue, and may affect physical function and level of abilities (Ahmad and Kim, 2018).

In order to evaluate and measure the effect of poor postures, advanced sensor technologies have been developed. Many sensors with corresponding signal-processing techniques have been used to investigate the fatigue process during physical activities. Examples include the motion capture-based sensors that can capture the kinematic features of humans (Bailey et al., 2018), accelerators with dynamic movements and the related metrics (Beato et al., 2019), surface electromyography (sEMG) sensors to measure the muscle activities (Edouard et al., 2018), etc. Among them, the sEMG signals are shown to have excellent ability on revealing muscle activity at any time instant during various movements and postures (Blanc and Dimanico, 2010). The sEMG signal can provide information to characterize muscle fatigue by means of changes in signal indicators (Marshall et al., 2018; Toro et al., 2019), including the mean absolute value (MAV) (Toro et al., 2019), root mean square (RMS) (Girard et al., 2018), mean frequency (MNF) and median frequency (MDF) (Bowtell et al., 2014), and so on. As the sEMG signal is usually noisy, other advanced signal processing techniques, such as the wavelet transform, have been used to get better time-frequency resolutions (Chowdhury et al., 2013).

With analyzing sEMG signal collected from back muscles, it was shown that keeping the poor posture with long time had impacts on the development of muscle fatigue (Jia and Nussbaum, 2018). For example, with 1-h typing task, the neck, and shoulder pain were observed significantly increased with forward head and thoracic kyphotic posture (Kuo et al., 2019); with 40 min poor posture sitting, the trunk flexion and metrics of localized muscle fatigue were significantly increased (Jia and Nussbaum, 2018).

However, it is not clear whether a short-duration of poor posture will have some negative impact on humans. It is known that the development of muscle fatigue is a time-varying procedure, it is challenging to detect muscle fatigue from the sEMG signal with a short-time duration of static poor posture, as changes are invisible due to small signal-to-noise ratio in sEMG signals. Although physiotherapists think that a short period of poor posture will also have some negative impact on people, there is a lack of systematic study to support this belief. As physical activities can somehow speed up the muscle fatigue process, this work tries to provide some evidence that sitting in poor posture indeed affects the performance of subsequent physical activities by carefully designed experiments. In this work, the push-up is selected to represent a common physical activities due to its simplicity.

The experiment consists of two steps. The first step measures the baseline of push-ups with two sEMG sensors to detect the fatigue of upper limb muscles. In the second step, 15-min of a poor sitting posture is followed by the same push-up activities. The duration of short-period poor posture is suggested by the physiotherapist. In the second step, each participant wears sEMG sensors on 8 back muscles. The location of each sEMG sensor is suggested and verified by a physiotherapist.

The hypothesis is that sitting in poor posture for a short time will affect the performance of the following physical activities. If this hypothesis is true, this suggests that some parts of back muscles are getting fatigued during the short period of poor posture, then some advanced signal processing techniques can be used to further investigate the measured sEMG signal from the back muscles.

The experimental results from 14 healthy male subjects support our hypothesis from the statistics of sEMG measurements. Our results indicate that poor posture, even a short-duration one, will have negative impact on human activities. Therefore, people should be always careful of avoiding poor postures.

## 2. Materials and Methods

### 2.1. Participants

Fourteen healthy male subjects were recruited for the experiment, the detailed information of the participants is shown in Table 1, including age, height, weight, and body mass index (BMI). All subjects were right-hand dominant. The participants were informed about the purpose and content of the experiment, and a written consent was obtained prior to the study. The project was approved by the Human Research Ethics Committee of the University of Melbourne (#1954575).

**Table 1:** Characteristic information of the participants.

	**Maximum**	**Minimum**	**Median**	**Mean ± SD**	***p*-value**
Age (y)	29	20	23	23.71 ± 2.49	0.132
Height (cm)	191	168	179	178.79 ± 6.30	0.973
Weight (kg)	99	66	76.25	79.75 ± 11.91	0.056
BMI (kg/m^2^)	29.26	21.31	25.26	24.86 ± 2.62	0.353

### 2.2. Experimental Setup

**Figure 1:**
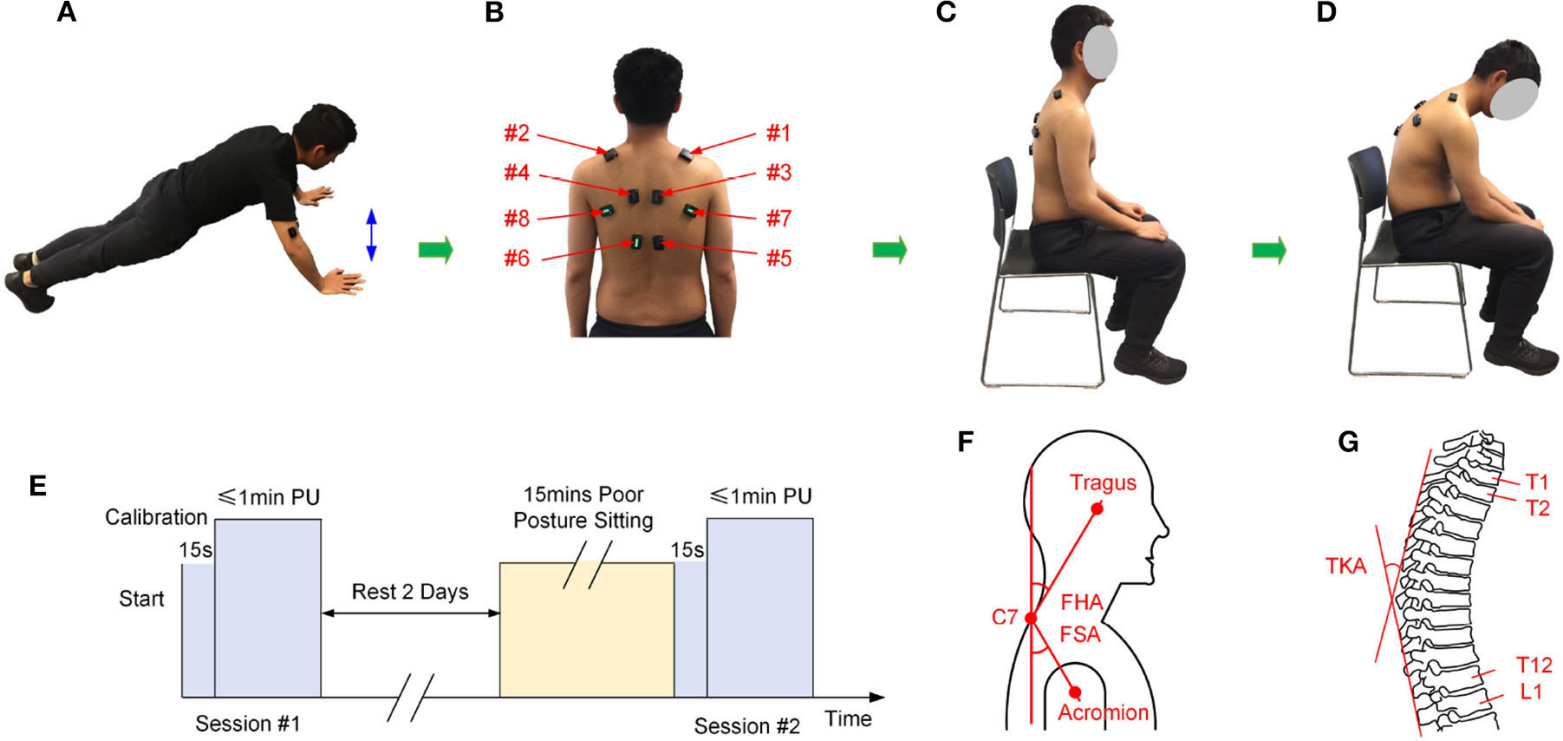
The experimental setup, **(A)** the physical exercise of push-up, **(B)** locations for the eight sEMG sensors, **(C)** illustration of the natural posture, **(D)** illustration of the poor posture, **(E)** the experimental procedure, **(F)** measurement of the FHA and FSA (Thigpen et al., 2010), **(G)** measurement of the TKA (Lewis and Valentine, 2010). PU, Push-up.

The procedures of experimental setup are illustrated in Figure 1. As shown in Figure 1A, the physical exercise of push-up is selected in our experiment as suggested by the physiotherapist. It is believed that similar performance can be observed if other physical activities are used.

As discussed in section 1, it is difficult to detect the small changes during poor posture sitting with a short time, the experiment consists of two sessions of push-ups (highlighted in Figure 1E) to evaluate the influence of poor posture. During the first session, the participants were instructed to finish the exercise of push-up in their normal condition. In this session, the participants are asked to do push-ups for 1 min or until the participants could not continue when it is less than 1 min. The sEMG sensors were calibrated before each experiment.

Before conducting the second experiment session, the participants were asked to have at least 2 days rest for muscle recovery, this was indicated from the literature that 97% of force production capacity had returned within 48 h with a strenuous heavy resistance exercise protocol (Lipshutz et al., 1993). Moreover, the recruited subjects were elite athletes and graduate students, they were young and quite active already, and no one reported delayed onset of muscle soreness (DOMS) before conducting the second phase exercise. During the second session, the participants were first instructed to sit with the poor posture, which was illustrated in Figure 1D: sitting with forward head, increased thoracic kyphosis, and rounded shoulder posture. The participants were advised to keep the poor posture for 15 min with minimal posture adjustments or in-chair movement. During the experiment, each subject was allowed to “free his hands,” for example, the subject can operate a mobile phone. After sitting with poor posture for 15 min, the participants were required to finish the 1 min push-up protocol as described in the first session.

The DELSYS Trigno Biofeedback System (DELSYS Inc., Natick, MA, USA) was used to collect sEMG signals for analysis. Two sources of sEMG signals were used during the experiment, (1) the sEMG signals were collected from biceps brachii and triceps brachii during push-up exercises in the two sessions. (2) During the poor posture sitting, sEMG signals were collected from eight upper back muscles, including the right upper fibers of trapezius (#1, RUFT), left upper fibers of trapezius (#2, LUFT), right middle fibers of trapezius (#3, RMFT), left middle fibers of trapezius (#4, LMFT), right lower fibers of trapezius (#5, RLFT), left lower fibers of trapezius (#6, LLFT), right infraspinatus (#7, RI), and left infraspinatus (#8, LI). Locations of the eight sensors was illustrated in Figure 1B. The location of each sEMG sensor was adjusted for each participant according to the suggestion of the physiotherapist. Meanwhile, three angles of the natural sitting and poor sitting postures were measured, including the forward head angle (FHA), the forward shoulder angle (FSA), and the thoracic kyphosis angle (TKA) (Lewis and Valentine, 2010; Thigpen et al., 2010). Illustration of the three angles were demonstrated in Figures 1F,G, and the measurements were shown in Table 2. The normality tests of the absolute difference of the angles were performed.

**Table 2:** Measurement of angles for the natural posture and the poor posture (Mean ± SD).

	**Natural posture**	**Poor posture**	**Absolute difference**	***p*-value**
FHA (°)	39.81 ± 8.31	78.74 ± 5.07	38.93 ± 9.80	0.658
FSA (°)	28.96 ± 2.66	15.70 ± 2.95	13.26 ± 1.89	0.140
TKA (°)	36.90 ± 6.23	59.39 ± 1.51	22.49 ± 5.49	0.124

### 2.3. Statistical Analysis

Standard statistical analysis was performed on the collected data. Prior to the statistical analysis, data normality was checked with the Shapiro-Wilk test. Depending on whether the data distribution is normal or not, paired *t*-test or Wilcoxon signed rank test was performed to compare the frequency changes of push-up exercise in the two experiment sessions. One-way Analyses of Variance (ANOVA) with repeated measures or Kruskal-Wallis test was employed to measure frequency changes among different levels (the 1st min, 5th min, 10th min, and 14th min) during the poor posture sitting. The Tukey *post hoc* test was used to investigate the significant difference between different levels. The significance value was set at 0.05, and the data was presented as mean value ± standard deviation.

## 3. Data Analysis and Results

### 3.1. Poor Posture Affected Upper Limb Muscle Fatigue

**Figure 2:**
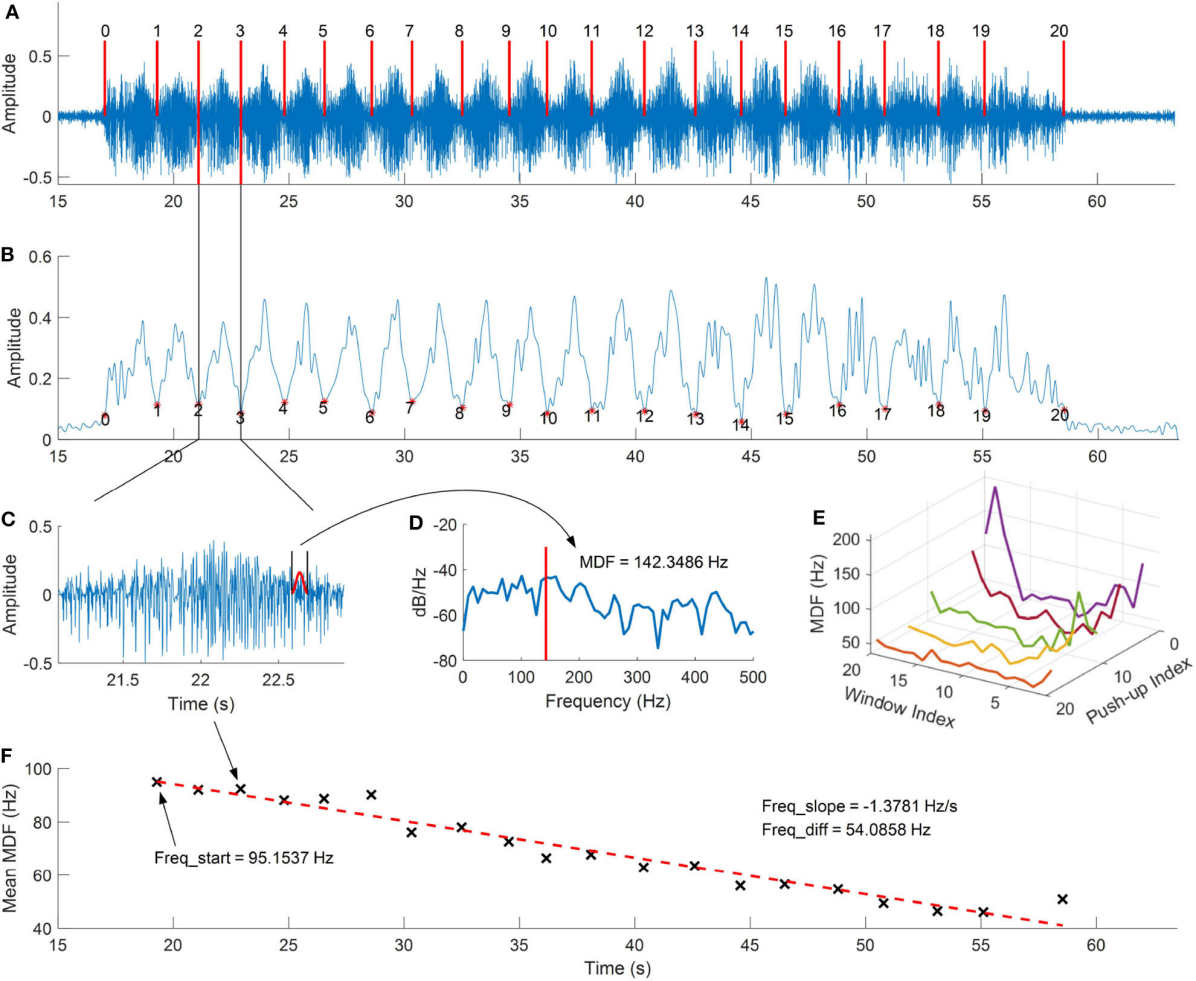
Procedures of the sEMG data analysis, **(A)** raw sEMG signal collected from the triceps brachii during push-up, **(B)** envelop analysis and push-up detection, **(C)** sEMG signal for a single push-up, **(D)** the spectral analysis for the moving hamming window segment, **(E)** median frequency curve of each push-up, **(F)** the mean median frequency and linear fitting of each push-up.

The signal processing procedures for the sEMG signal collected from upper limb muscles during the push-up protocol are shown as Figure 2. The raw sEMG signal recorded at triceps brachii during the push-up protocol is shown as Figure 2A. The signal is sampled with 2,148 Hz and some standard signal processing techniques were applied to pre-process this signal. These include outlier removal (three standard deviations from the mean value) and filtering (the 4th order Butterworth band-pass filter with the frequency range of 10–500 Hz). The filtered signal is then used for envelope analysis and separating bursts of muscle activity during each push-up. The procedures are as following, first, the RMS is calculated to smooth the signal with time span of 100 ms; secondly, the envelope analysis is carried out to find the local minimal value of the RMS signal, and the valid burst separation points are obtained by comparing the identified local minimal points with a threshold, which can be roughly estimated by the time duration of burst, in the present study, the threshold value is usually set as 1 s depending on each participant. The envelope analysis and identified bursts of push-up are shown as Figure 2B, it can be seen from the figure that bursts corresponding to a total of 20 push-ups are efficiently identified.

The MDF and MNF are then calculated for each burst, the Hamming window with length of 100 ms is used to obtain each segment in sEMG signal, the envelope of the windowed signal is illustrated as Figure 2C. Then the power spectral analysis for the segment is calculated as Figure 2D, and the curve of MDF for each burst can be obtained by moving the Hamming window, which is illustrated as Figure 2E and the mean value of each curve is calculated for regression analysis. As shown in Figure 2F, the linear regression is used to fit the calculated MDF values, and the frequency information is used for statistical analysis, including the slope of the linear curve, the difference between the start and end frequencies, and the frequency difference percentage, which is calculated as the difference divided by the start frequency.

**Figure 3:**
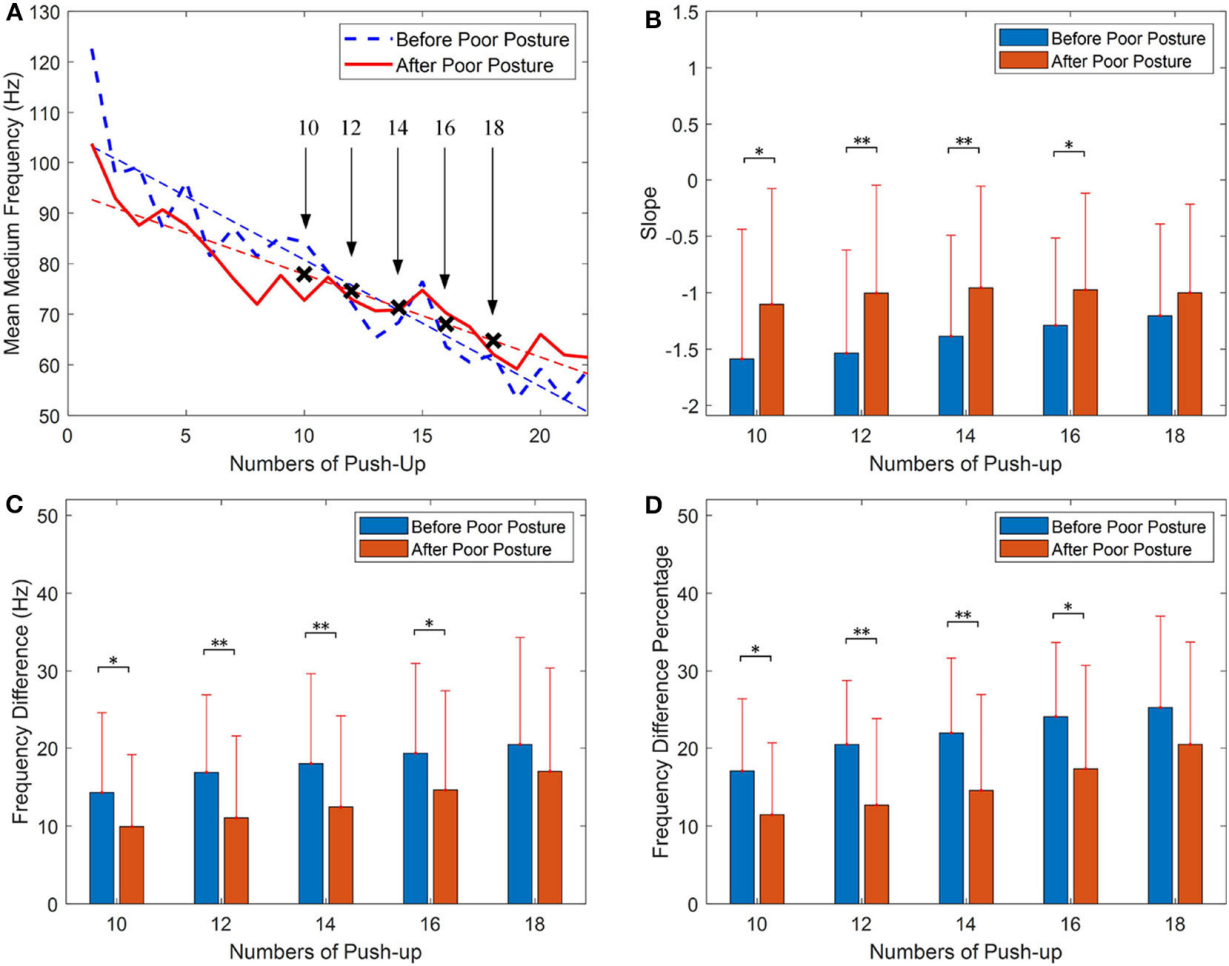
Fatigue analysis of push-up with median frequency (MDF), **(A)** comparison of linear regression before and after the poor posture, **(B)** statistical analysis of the slope values, **(C)** statistical analysis of the differences between the start frequency and the end frequency, **(D)** statistical analysis of the frequency difference percentage (the data is presented as mean value ± standard deviation; *p < 0.05, **p < 0.01).

The fatigue analysis with the mean MDF for push-up before and after the poor posture protocol is demonstrated as Figure 3. It can be seen from Figure 3A that the slopes of the linear curves before and after poor posture are −2.506 and −1.3721, respectively. This result indicates the muscle fatigue has a slower decreasing trend after the poor posture. Statistical analysis of the slope values with increasing push-up numbers is shown in Figure 3B, it can be seen from the figure that the slopes of the regression curves before poor posture are smaller than that after poor posture, while with push-up numbers of 10, the mean slopes of the regression curves before poor posture are significantly (*p* = 0.025) smaller than those after poor posture. The statistical analysis of the difference value between the start and the end frequency is shown as Figure 3C, it can be seen from the figure that the mean value of the frequency difference before poor posture is larger than that after poor posture. Figure 3D represents the statistical analysis for the percentage value of the frequency difference with a significant value obtained with 12 push-ups (*p* = 0.002), which indicates that the muscle fatigue process has a smaller decreasing percentage after poor posture.

**Figure 4:**
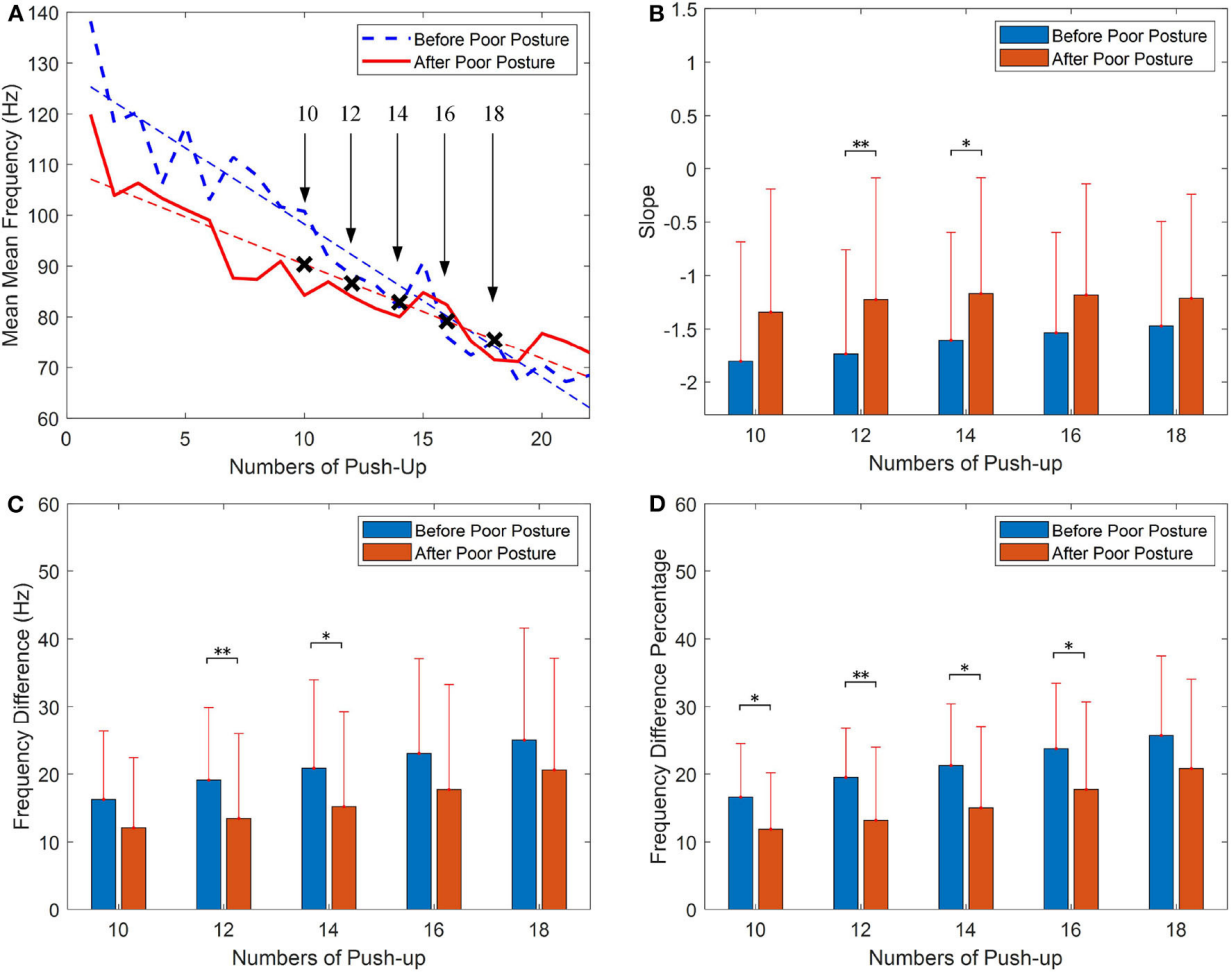
Fatigue analysis of push-up with mean frequency (MNF), **(A)** comparison of linear regression before and after the poor posture, **(B)** statistical analysis of the slope values, **(C)** statistical analysis of the differences between the start frequency and the end frequency, **(D)** statistical analysis of the frequency difference percentage (Presented as mean value ± standard deviation; *p < 0.05, **p < 0.01).

Similar results were obtained by calculating the mean MNF values of the triceps brachii. Figure 4 demonstrated the statistical analysis of frequency information of the mean MNF values. As shown in Figure 4A that the slope value of regression curve obtained for push-up before poor posture and after posture are −3.013 and −1.589. Figure 4B shows the comparison of mean slope values obtained for the two groups, the slope values obtained for 12 and 14 push-ups before poor posture are significantly (*p* = 0.007; *p* = 0.020) smaller than those obtained after poor posture. The similar comparison can be found in Figure 4C. The comparison of statistical analysis for the percentage value of the frequency differences is shown as Figure 4D, this figure shows that the mean percentage values of the frequency differences before poor posture are smaller than those after the posture protocol.

### 3.2. Upper Back Muscle Fatigue Analysis

It can be seen from section 3.1 that significant differences are observed for the push-up before and after the short-time poor posture sitting in terms of the frequency change in mean MDF and MNF, which indicates the poor posture has influence on the performance of physical exercise of the group. In this section, a detailed investigation of upper back muscles related to the poor posture is presented to evaluate the changes and sensitive frequencies of upper back muscles during the poor posture sitting.

**Figure 5:**
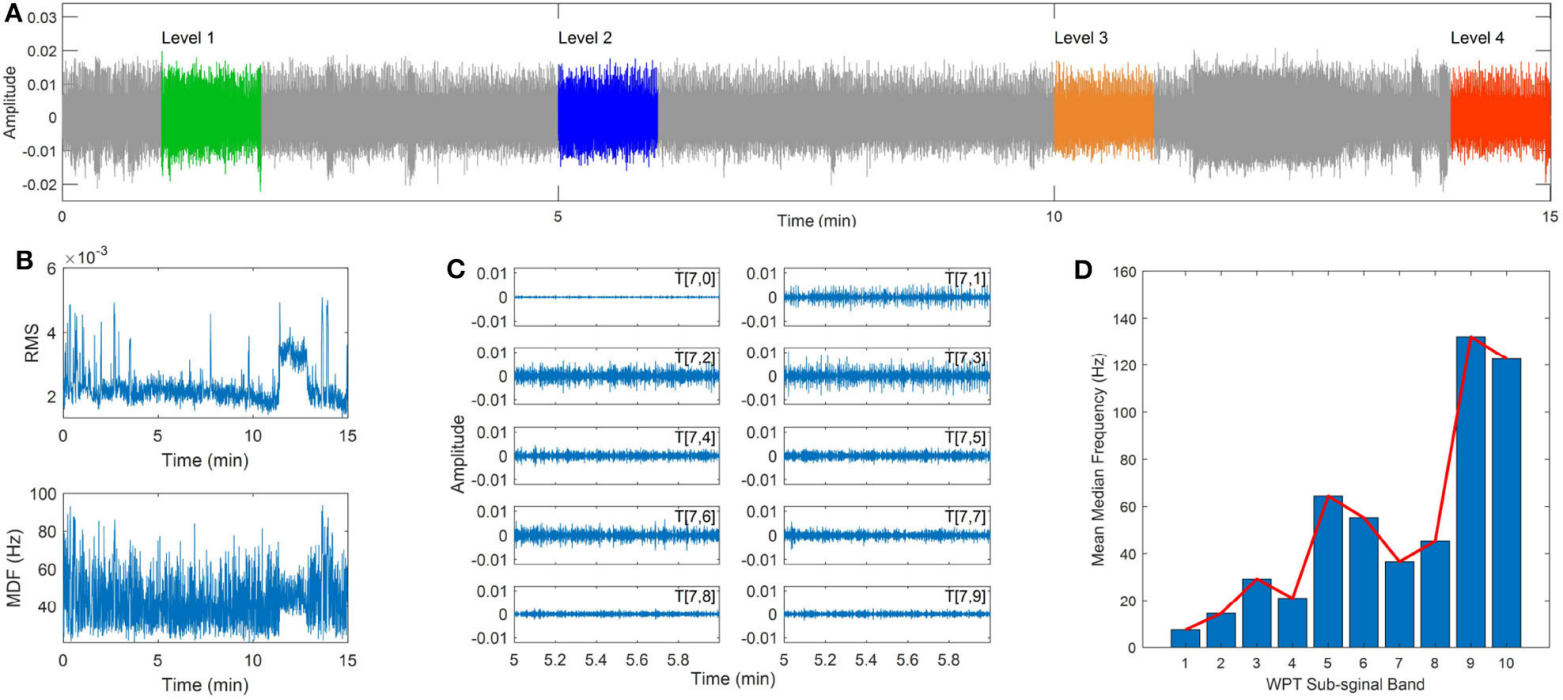
sEMG signal collected during static sitting and wavelet analysis, **(A)** the raw sEMG signal, **(B)** the RMS and median frequency features of the sEMG signal, **(C)** the sub-signal obtained from WPT decomposition, **(D)** the mean median frequency of each sub-signal.

As described in the experiment setup, eight wireless sensors are employed to collect sEMG signal from upper back muscles during the poor posture sitting. An illustration of the raw sEMG signals collected from the LLFT muscle during the 15 min poor posture sitting is demonstrated in Figure 5A. The widely used RMS and MDF features are calculated from the sEMG signal after band-pass signal filtering, which are time and frequency domain features respectively. It can be seen from Figure 5B that there is heavy noise, and significant fluctuation in the two features, therefore only limited information can be obtained from the two features.

As indicated in previous studies (Chowdhury et al., 2013; Varrecchia et al., 2020), the wavelet transform (WT) was a powerful technique in detecting small changes in a noisy signal. The WT technique can obtain high resolution in the analysis of sEMG signal by decomposing the raw signal into a series of sub-signals with different frequency bands. Similar to the WT, the wavelet packet transform (WPT) is a generalization of the WT and can obtain both low and high frequency information of the sEMG signal (Hekmatmanesh et al., 2019).

The WPT analysis involves two parameters, the wavelet function and the number of levels into which the signal will be decomposed. In the present study, the widely used wavelet families are employed for the sEMG signal analysis, including the Daubechies (db) wavelets, the Symlets (sym) wavelets, the Biorthogonal (bior) wavelets, and the Coiflets (coif) wavelets. The decomposition level was set as 7 to obtain a fine resolution in the frequency domain (Kilby and Hosseini, 2004; Chowdhury et al., 2013). The previous 10 sub-signals of the original sEMG signal obtained by the WPT method is shown in Figure 5C, and the corresponding median frequency of each sub-signal is demonstrated in Figure 5D. It can be seen that the median frequency indicates a gradually increasing trend with increasing the index number of the sub-signal.

**Figure 6:**
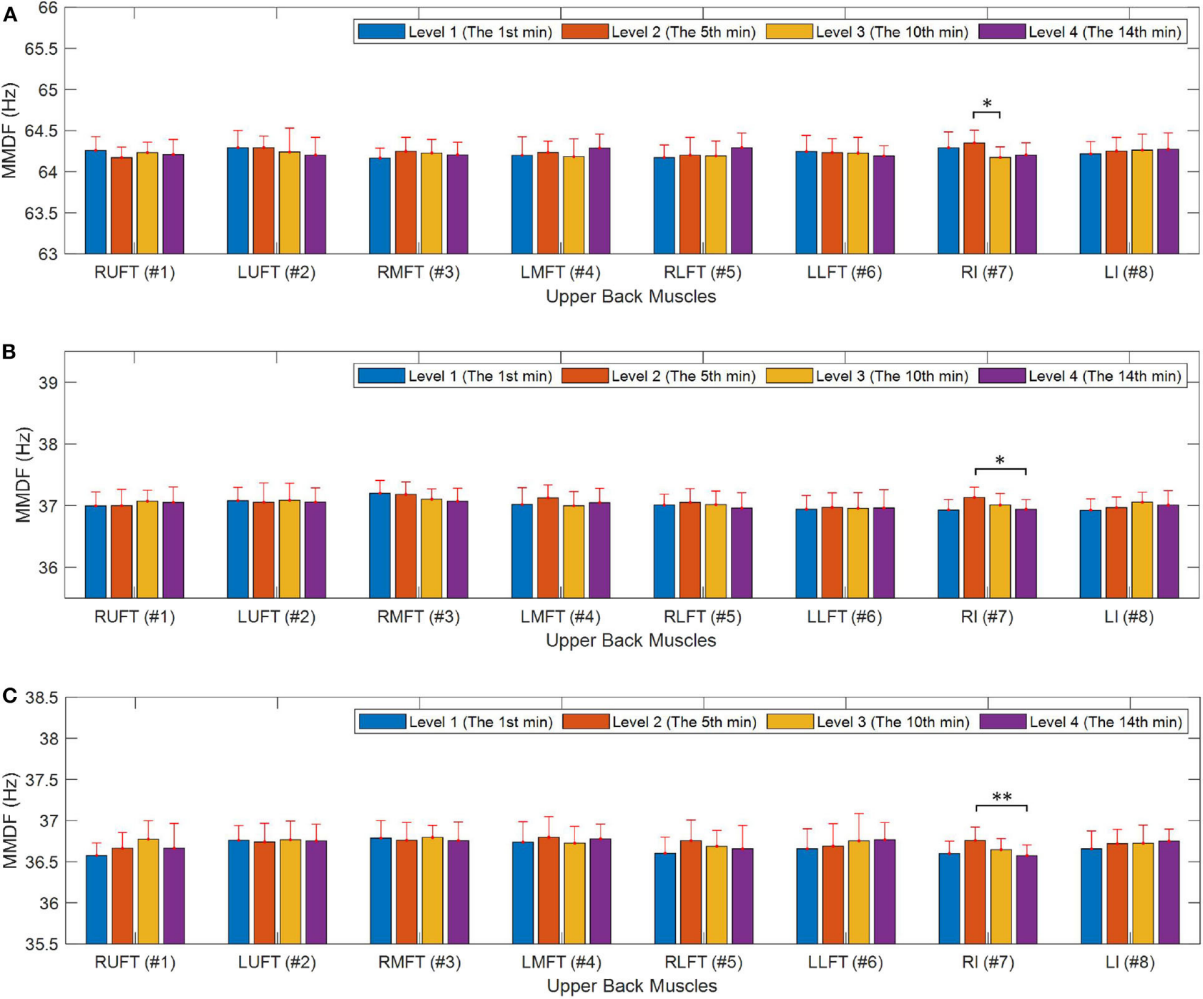
Upper back muscle fatigue analysis during the poor posture, **(A)** muscle fatigue analysis in the 5th sub-signal with the coif4 wavelet, **(B)** muscle fatigue analysis in the 7th sub-signal with the coif4 wavelet, **(C)** muscle fatigue analysis in the 7th sub-signal with the coif2 wavelet. *p < 0.05, **p < 0.01.

Four segments of the raw sEMG signal in the 1st min, 5th min, 10th min, and 14th min were used for the comparison study, which were denoted as level 1 to level 4. The WPT technique is used to extract sub-signals from the raw sEMG signal, and a moving window is applied to obtain the frequency spectrum for each sub-signal with the length of 1 s and the overlap rate of 0.5. The median frequency was calculated for each window frame and the mean value of median frequencies (MMDF) was calculated for all windows. Figure 6 demonstrates the calculated MMDF for all eight upper back muscles at the four time levels in different wavelet functions and sub-signals.

It can be seen from Figure 6 that significant differences were observed for the RI muscle between four time levels in all the three sub-figures. Specifically, as demonstrated in Figure 6A, with the coif4 wavelet, the MMDF value of level 3 in the 5th sub-signal is observed smaller than the value of level 2, which is around 64 Hz (*p* = 0.024). As demonstrated in Figure 6B, the MMDF value of level 4 in the 7th sub-signal is smaller (*p* = 0.023) than the value of level 2 around 37 Hz. The similar frequency differences can be observed in Figure 6C, where the MMDF value of level 4 in the 7th sub-signal obtained by the coif2 wavelet is smaller than the value of level 2 around 36.5 Hz (*p* = 0.0095).

## 4. Discussion

There are two major findings in this study, first, the short-time poor posture indeed has an effect on the performance of physical exercise. Comparing the subjects' performance of push-ups before and after poor posture, the median frequencies of the two sessions have significant differences after 10 push-ups in terms of the slope of the trend (*p* = 0.025), the frequency differences between the start and the end of the push-up (*p* = 0.025), and the rate of the frequency differences (*p* = 0.017). Second, it is confirmed from experiments and data analysis from sEMG sensors, some parts of back muscles are affected during the short time of poor posture with a clear indication of fatigue, which has not been observed in literature. As indicated in Figure 6, the median frequency of RI muscle is observed to have significant differences between different time levels.

It is worthwhile to note that the obtained results using median frequency of sEMG signal collected from upper back muscles indicate that the wavelet transform especially the coif wavelet function has excellent performance on identifying the small frequency changes when signal-to-noise ratio is small. Moreover, our analysis shows that sEMG signals of each muscle has an active frequency range. As shown in Figure 6, the median frequencies of the RI muscle around 36 and 64 Hz are more sensitive. This finding is consistent with that in previous research (Chowdhury et al., 2013), which indicated that the lower frequencies of the sEMG signal are more sensitive to fatigue induced by dynamic repetitive exertions.

The study aims to enhance the understanding of the effects of poor posture on muscular mechanics. Previous studies indicated that the poor posture may require larger muscle force to counterbalance external moment, and even small elevations of cervical extensor activities could result in cumulative effects such as muscle fatigue and other neuromuscular alternations (Ning et al., 2015). The extensor muscles and posterior ligaments of the dorsal spine are stretched with the increased forward head and kyphosis position (Sun et al., 2014; Singla and Veqar, 2017). It should be noted that fascia has been described as a body-wide tensional network, it consists of all fibrous collagenous soft connective tissues, the body-wide “fascial net” allows for multi-articular tensional strain transmission (Findley and Schleip, 2007; Schleip and Müller, 2013). Especially, Dr. Yoshitake's work presented a strong evidence to support the presence of epimuscular myofascial force transmission (EMFT) in upper extremity muscles, and indicates that the EMFT may be facilitated at stretched muscle lengths [6]. With the existence of myofascial chain, the strain in the overstretched back muscles by the poor sitting posture may affect some parts interconnected muscles of the upper limb (Wilke et al., 2017; Yoshitake et al., 2018). They are forced to maintain such posture and would affect the force production of those parts. There are effects on circulation, and these factors should reflect adversely on subsequent exercise.

The effects of myofascial force transmission on adjacent synergistic muscles have been further studied by the finite element modeling (Yucesoy et al., 2003a; Pamuk et al., 2020). These studies suggest that the myofascial force transmission has major effects on skeletal muscle mechanics. More importantly, the changes of muscle relative position play an important role in the change of production of muscle force, because they affect the distributions of sarcomere lengths, which are the key determinants of muscle active force production (Yucesoy et al., 2003b, 2006). These available results would support to explain our observations of the difference of muscle function before and after the poor sitting posture in the present study, as the poor sitting posture in the present study changes the muscle relative position to the surroundings, which in return alters the sarcomere length distributions and results in an altered muscle function.

The study has important implications in both biomechanical and clinical settings. First, the results indicate that though the poor posture is defined as forward head posture, rounded shoulders, and increased thoracic kyphosis, the process of maintaining such a posture may have effects on other regions of muscles, the impact of poor posture should be evaluated comprehensively; Second, the mentioned short-duration poor posture is quite common in real life, for example, it might correspond to a sports break between two consecutive games. Another possible scenario is using one's phone on the bus or train. The finding in this paper indicates the possible impact on athletes specifically during their preparation for optimal performance, as well as to the general population on the effect of poor posture. The results suggest that short-duration poor posture might lead to muscle fatigue of some parts of the back muscles, and the performance of physical activities will be degraded (immediately and with latent effects) after the poor posture. People should be mindful of their postures if they will undertake physical activities in the near future.

The current study has some limitations. First, only male subjects were recruited for the study, as the experiment requires placing eight sensors on the back muscles, due to the privacy issue and lack of female team members to collect data from female volunteers. We will further investigate whether there has gender difference for the results. Second, due to the limitation of available data, the current study does not include the results from natural posture. The next step research will recruit more subjects to evaluate the effect of poor and natural postures, and investigate the prevention to the immediate and latent effects of muscle fatigue from the poor posture, which will provide know-how and awareness to prevent during sports and most importantly the activities of daily living (ADLs).

## 5. Conclusions

This study contributed to the understanding of a particular short-duration poor sitting posture on the effect of the performance of physical exercise and the fatigue of back muscles. From statistics of 14 subjects, it was observed that the median frequencies of the sEMG signal of the triceps brachii has significant differences after poor sitting posture, and the median frequencies collected from the RI muscle in the upper back have been shifted in different time levels. The results suggest that the poor posture will lead to a quicker fatigue procedure of the following physical exercise as shown in the median frequency of the corresponding muscles. The results also demonstrated that the proposed frequency analysis techniques were able to identify the frequency range of the individual muscle, which can be used to show a clear shift of median frequencies of noisy sEMG signals in order to represent fatigue.

## Data Availability Statement

The datasets that support the findings of this study are available from the corresponding author upon reasonable request.

## Ethics Statement

The studies involving human participants were reviewed and approved by the Human Research Ethics Committee of the University of Melbourne (#1954575). The patients/participants provided their written informed consent to participate in this study.

## Author Contributions

LL, XG, MR, KG, and YT contributed the experiment design and data collection. LL and YT contributed the data analysis and drafting the manuscript. LL and MR performed the statistical analysis. KG, IM, and DO contributed the manuscript revision and results interpretation. All the authors read and approved the manuscript.

## Conflict of Interest

IM is employed by the IBM Research - Australia. KG is a sport physiotherapist for the Melbourne University Elite Athlete Unit, and CEO of the Elite Akademy Sports Medicine. The remaining authors declare that the research was conducted in the absence of any commercial or financial relationships that could be construed as a potential conflict of interest.
